# Epidermal Immunity and Function: Origin in Neonatal Skin

**DOI:** 10.3389/fmolb.2022.894496

**Published:** 2022-06-08

**Authors:** Marty O. Visscher, Andrew N. Carr, Vivek Narendran

**Affiliations:** ^1^ James L. Winkle College of Pharmacy, University of Cincinnati, Cincinnati, OH, United States; ^2^ The Procter and Gamble Company, Cincinnati, OH, United States; ^3^ Perinatal Institute, Cincinnati Children’s Hospital Medical Center, Cincinnati, OH, United States

**Keywords:** epidermal barrier, immunity, stratum corneum, neonatal, skin, proteomics, genomics, vernix caseosa

## Abstract

The fascinating story of epidermal immunity begins *in utero* where the epidermal barrier derives from the ectoderm and evolves through carefully orchestrated biological processes, including periderm formation, keratinocyte differentiation, proliferation, cornification, and maturation, to generate a functional epidermis. Vernix caseosa derives from epidermal cells that mix with sebaceous lipids and coat the fetus during late gestation, likely to provide conditions for cornification. At birth, infants dramatically transition from aqueous conditions to a dry gaseous environment. The epidermal barrier begins to change within hours, exhibiting decreased hydration and low stratum corneum (SC) cohesion. The SC varied by gestational age (GA), transformed over the next 2–3 months, and differed considerably versus stable adult skin, as indicated by analysis of specific protein biomarkers. Regardless of gestational age, the increased infant SC proteins at 2–3 months after birth were involved in late differentiation, cornification, and filaggrin processing compared to adult skin. Additionally, the natural moisturizing factor (NMF), the product of filaggrin processing, was higher for infants than adults. This suggests that neonatal skin provides innate immunity and protection from environmental effects and promotes rapid, continued barrier development after birth. Functional genomic analysis showed abundant differences across biological processes for infant skin compared to adult skin. Gene expression for extracellular matrix, development, and fatty acid metabolism was higher for infant skin, while adult skin had increased expression of genes for the maintenance of epidermal homeostasis, antigen processing/presentation of immune function, and others. These findings provide descriptive information about infant epidermal immunity and its ability to support the newborn’s survival and growth, despite an environment laden with microbes, high oxygen tension, and irritants.

## 1 Introduction

Epidermal immunity is prominent in the major global health issue of high neonatal mortality due to sepsis. Among nearly three million live births in 14 countries from 1979–2019, there were 29,608 sepsis cases, corresponding to 2,842 in 100,000 live births and 17.9 percent mortality (Fleischmann et al., 2021). Unfortunately, the rate was 1.4 times higher for the most recent decade (2009–2018). The cost of neonatal sepsis was 469 billion US dollars, as of 2014 (Ranjeva et al., 2018). Skin-based infant care practices, including kangaroo mother care where there is uninterrupted contact with the infant and mother (chest to chest) and only breastfeeding for nutrition (Conde-Agudelo and Diaz-Rossello, 2016), newborn umbilical cord treatment with chlorhexidine (Imdad et al., 2013), and repetitive application of topical emollients, such as sunflower oil, in hospitalized premature infants (Darmstadt et al., 2004; Darmstadt et al., 2005; Darmstadt et al., 2008; Darmstadt et al., 2014), have reduced infection rates compared to no intervention.

Despite advances in medical care, premature birth remains high, at about 11% of births worldwide (WHO, 2012). Late-onset sepsis is the cause of mortality and morbidity in this population (Stoll et al., 2002). Clinical practice changes, specifically the implementation of evidence-based catheter insertion practices in 22–29 weeks gestation infants, have reduced catheter-related bloodstream infections significantly, but to a lesser degree than expected (Kaplan et al., 2011). Poor skin integrity may be a major predisposing factor for neonatal sepsis. The development of interventions to enhance stratum corneum (SC) formation is a global priority (Lawn et al., 2006) and essential for reducing the consequences of epidermal barrier immaturity (Rutter, 1996; 2000).

In this review, we discuss epidermal immunity from its origins, namely during fetal development, late gestation, at birth, and over the first few months of life. The inherent benchmark for epidermal immunity is adult epidermis, viewed as a stable, steady-state condition. The intrinsic self-renewal feature distinguishes the skin and, thereby, the dependence on the provision of continual immune function. The newborn infant provides a truly unique opportunity to observe its rapid evolution, namely, during adaptation from a water-based vessel to the gaseous, somewhat hostile terrestrial environment. We consider the role of vernix caseosa in this process and investigate epidermal immunity from the origin, using proteomic and genomic techniques and discuss the implications for improving clinical outcomes.

## 2 Fetal Skin Development

A single epithelial layer forms from ectoderm during embryogenesis under the influence of fibroblast growth factors, bone morphogenic proteins, and Notch signaling (Fuchs, 2007). A basal epidermal layer and one periderm layer have been created by gestational week 4 (Lane, 1986). The periderm covers the basal layer and forms tight junctions during fetal development. Melanocytes appear in the basal layer during weeks 5–8. Three epidermal layers appear by weeks 8–11. Proliferation and maturation of basal keratinocytes produce the spinous layer beneath the periderm and begin to stratify (Koster and Roop, 2007) (Blanpain and Fuchs, 2009). Four to five epidermal layers appear throughout gestational weeks 16–23. When the periderm regresses around week 23, fetal suprabasal cells adhere to other cells to create a barrier structure (Sumigray and Lechler, 2015). By 26 gestational weeks, the epidermis consists of one basal layer, 2-3 spinous layers, one granular layer, and 5-6 stratum corneum layers (Holbrook and Odland, 1975). Eight distinct phases of differentiation occur over gestational weeks 5 and 26 (Holbrook and Odland, 1975).

Hair follicles begin to form throughout weeks 9–14. In the second of eight phases, the epidermis begins to thicken in certain regions, giving rise to hair pegs (stage 3) (Hu et al., 2018). Fibroblasts from the dermis collect at the lower end of the peg to generate a sphere-shaped dermal papilla. The root sheath moves up the hair follicle and the follicle grows in a downward direction in stage 6. The hair extends out through the skin surface in stage 6. The sebaceous glands develop near the upper hair follicle at gestational weeks 13–14. Eccrine glands develop at about the same time and continue to develop through gestational week 24 (Fu et al., 2005).

Leukocytes that are positive for the human leukocyte antigen dendritic major histocompatibility complex cell surface receptor (HLA-DR) develop in the fetus about gestational week 5 and in the skin at week 7 (Schuster et al., 2009). Predecessors of Langerhans cells (LC) emerge and produce antigens at gestational weeks 7–9 (Foster et al., 1986; Fujita et al., 1991). Adult LC is characterized by the presence of Birbeck granules, CD207/langerin, and CD1a but these features are not seen in the fetus until gestational week 11 (Foster et al., 1986; Schuster et al., 2009). Mast cells appear only after gestational week 11 and then increase in the second trimester (Schuster et al., 2012).

## 3 Birth

Few events are as dramatic and “life changing” as birth, when the human infant abruptly transitions from warm, wet, nurturing, serene *in utero* conditions to a cooler, dry, gaseous, microbe laden environment. The infant immediately relies on a robust innate immune system, provided by the epidermal barrier, and begins self-sufficiency with air-breathing, nutrient intake, and growth. Epidermal differentiation generates the stratum corneum (SC), the essential innate immune interface between the living infant and the external environment. For the full-term infant, the SC provides 1) a barrier to water loss from within and irritants from outside, 2) thermal regulation, 3) sensation and tactile discrimination, 4) an acid mantle, 5) immunosurveillance and infection control and 6) tractability to mechanical trauma.

This extraordinary process begins from ∼5 to 26 weeks gestation when the periderm shields the epidermis from amniotic fluid. At ∼ 23.5 weeks, the periderm is no longer present and keratin-containing cells are noted in the interfollicular spaces and along the hair follicle (Holbrook and Odland, 1975). Starting at weeks 18–19, the stratum corneum barrier forms, that is, cornification of epidermal corneocytes takes place. Initially, SC formation occurs around/along the hair follicle, then on the head (week 23), and later on the abdomen (week 25) (Hardman et al., 1999).

## 4 Vernix Caseosa

### 4.1 Vernix Origins and Formation

Histological and microscopic examination of vernix caseosa found ovid or polygonal cells without nuclei or organelles while some had nuclear ghosts (Agorastos et al., 1988; Pickens et al., 2000). The cellular acid phosphatase activity was variable, from none to very high. The cytoplasm and cell membranes showed no alkaline phosphatase activity. The cells were thin (1–2 μm) and differed from regular to irregular with 5-6 sides with microvilli projections on the surface. The degree of keratinization varied, suggesting that they were from the outermost fetal stratum corneum and deeper levels, that is, less mature keratinocytes. Alternatively, the cells may originate during the transition from periderm to keratinized epidermis. There were few keratin filaments, lacking in orientation, and no evidence of desmosomes. In micrographs, the lipids between cells were generally amorphous, with occasional lamellae (Pickens et al., 2000). The cell diameter was ∼40 μm, larger than stratum corneum cells, perhaps due to absorption of water from amniotic fluid, and individual cellular hydration varied (Pickens et al., 2000).

Vernix caseosa is an amorphous, white waxy mixture of water-containing cells covered by a mixture of lipids (Pickens et al., 2000; Rissmann et al., 2006). It may appear on the fetal eyebrows at gestational week seventeen. Over time, it covers the fetal skin surface, advancing from head to toe and back to front (Visscher et al., 2005). Placental or hypothalamic corticotropic-releasing factors (CRF) may signal the pituitary gland to release adrenocorticotropic hormone (ACTH), causing the adrenal gland to release androgenic steroids (Zouboulis et al., 2003). They become active androgens and function within the sebaceous gland. Hair follicles have a local hypothalamic-pituitary-adrenal-like axis (Ito et al., 2005) that may be involved in vernix formation. Several vernix lipid types are also produced by the sebaceous glands, namely triglycerides, wax esters, and squalene (Nicolaides et al., 1972; Rissmann et al., 2006). Fetal cells possibly originate from the hair follicles (Kurokawa et al., 2009), mix with sebaceous lipids, extrude through the hair shaft, and continue to form and spread over the interfollicular epidermis during latter gestation (Hardman et al., 1998). Vernix cells may also come from the infundibular portion of sebaceous glands (Rissmann et al., 2006). Vernix films (i.e., spread on a porous substrate) *in vitro* are hydrophobic, due to the lipid cover on the hydrated cells (Youssef et al., 2001).

Vernix lipids cover the hydrated vernix cells to create a hydrophobic coating during latter gestation, thereby protecting the underlying fetal epidermis from exposure to amniotic fluid (Youssef et al., 2001). Vernix films are non-occlusive and permit water vapor transport through them (Tansirikongkol et al., 2007a). *In utero*, cornification of the fetal epidermis is incomplete thereby permitting a high water flux potential driven by osmotic gradients. Water gradients occur in skin homeostasis. Specifically, the SC exhibits a water gradient with higher hydration in the lower layers and decreased hydration at the skin surface (Warner et al., 1988; Caspers et al., 2003; Verdier-Sevrain and Bonte, 2007). The ability of the SC barrier to recover after the damage is due in part to the transepidermal water gradient and subsequently increased synthesis of DNA and lipids (Proksch et al., 1991; Denda et al., 1998a; Denda et al., 1998b; Fluhr et al., 1999). Vernix may serve as a semi-regulated barrier and/or physiological gradient for transepidermal water and nutrients *in utero*. This process, in turn, prompts epidermal cornification by increasing the synthesis of DNA and lipids.

As full-term gestation approaches, the mature fetal lungs secrete phospholipid surfactants that cause some of the vernix to detach from the skin surface (Narendran et al., 2000). This process causes the amniotic fluid to become cloudy. The infant swallows the amniotic fluid and, thereby, vernix provides nutrients to prepare the intestine for extra-utero feeding.

### 4.2 Vernix Composition and Function

**FIGURE 1:**
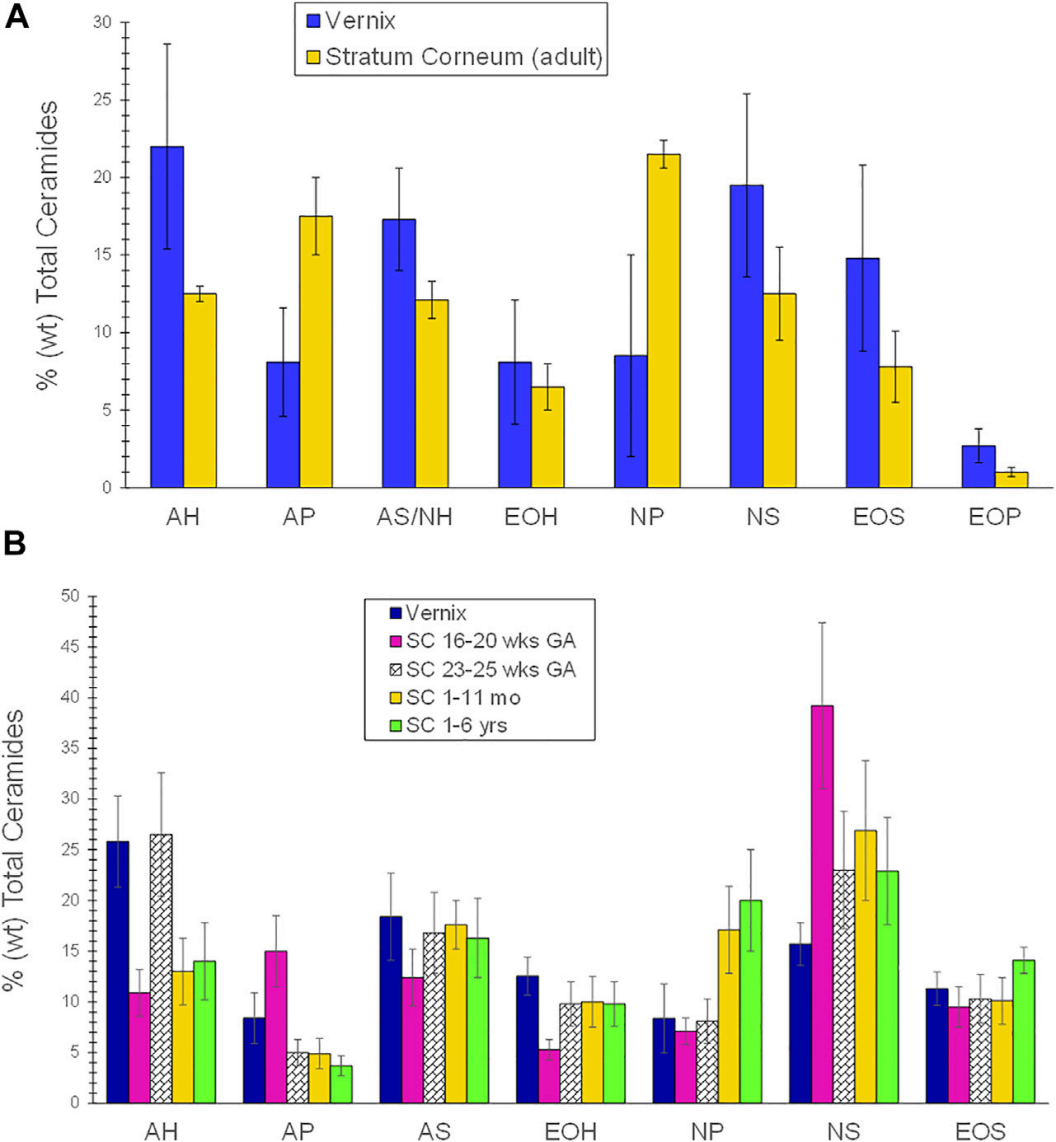
**(A)** A comparison of the ceramide profile of vernix caseosa and adult stratum corneum. The ceramide profile of vernix was compared to that of adult stratum corneum. Samples of vernix caseosa were collected at birth from full-term infants and adult tissues were obtained during cosmetic surgery (Rissmann et al., 2006). The lipids were extracted, separated by high-performance thin layer chromatography, and quantified. Values are given as percent weight as mean and ± standard deviations. Ceramide AH (AH contains α-hydroxy acids and sphingosines) was the most abundant, followed by NS (NS contains non-hydroxy fatty acids and sphingosines), AS/NH (AS contains α-hydroxy fatty acids and sphingosines and NH contains non-hydroxy fatty acids and 6-hydroxy sphingosine and EOS (EOS contains ester-linked fatty acids, ω-hydroxy fatty acids, and sphingosine). The relative ceramide levels were higher in vernix compared to adult SC except for ceramides AP (AP contains α-hydroxy fatty acids and phytosphingosine) and NP (NP contains non-hydroxy fatty acids and phytosphingosine) that were lower in vernix. The ceramide distributions were similar in vernix and adult SC. Statistical comparisons were not reported. **(B)** A comparison of the ceramide profiles in vernix, fetal stratum corneum (16–20 weeks GA), mid-gestational SC (23–25 weeks GA), infant SC (1–11 months), and child SC (1–6 years). Vernix caseosa from healthy full-term infants, tissue samples that required surgery, and fetal tissue from spontaneous abortions were quantified by high-performance thin layer chromatography (Hoeger et al., 2002). Ceramide (AH) was the highest fraction, followed by AS, NS, EOS, and EOH, with AP and NP being the lowest species. Vernix ceramide AH was significantly higher and vernix NP and NS were significantly lower than for infants of 1–11 months (*p* < 0.05). Ceramide levels in vernix and premature infant stratum corneum at 23–25 GA were comparable, except for ceramide AP that was higher in vernix (*p* < 0.05).

Vernix is composed of ∼80% water, associated with the cells, 10.3% protein, and 9.7% lipids (Pickens et al., 2000; Hoath et al., 2006). The non-lamellar lipid mixture that covers the flattened vernix cells comprises both non-polar, as the predominant fraction, and polar lipids, including fatty acids, ceramides, squalene, cholesterol, wax esters, and triglycerides (Rissmann et al., 2006). Epidermal barrier lipids, that is, cholesterol, fatty acids, and ceramides compose 10–30% of the vernix lipid fraction (Hoeger et al., 2002; Rissmann et al., 2006), with ceramide lipids comprising 4.9% of the total vernix lipid fraction (Rissmann et al., 2006). Vernix ceramide profiles (weight percent of total ceramides) compared to adult stratum corneum (SC) ceramides are in Figure 1A (Rissmann et al., 2006). Ceramide AH (AH contains α-hydroxy acids and sphingosines) was the most abundant in vernix, followed by ceramide NS (NS contains non-hydroxy fatty acids and sphingosines), ceramide AS/NH (AS contains α-hydroxy fatty acids and sphingosines and NH contains non-hydroxy fatty acids and 6-hydroxysphingosines) and ceramide EOS (EOS contains ester-linked fatty acids, ω-hydroxy fatty acids, and sphingosine). The distributions were similar while relative ceramide levels were higher in vernix compared to adult SC, except for ceramides AP (AP contains α-hydroxy fatty acids and phytosphingosine) and NP (NP contains non-hydroxy fatty acids and phytosphingosine) that were lower in vernix. The ceramide profiles in vernix, fetal SC (16–20 weeks GA), mid-gestational SC (23–25 weeks GA), infant SC (1–11 months), and child 1-6 years were compared, shown in Figure 1B (Hoeger et al., 2002) and the relative abundance of ceramides was similar to that of Rissmann (Rissmann et al., 2006). The vernix ceramide AH level was higher and vernix NP and NS levels were lower than those of infants aged 1–11 months (*p* < 0.05). Ceramide levels in vernix and premature infant stratum corneum at 23–25 gestational weeks were comparable, except for ceramide AP that was higher in vernix (*p* < 0.05). Collectively, these results reveal the uniqueness but also the similarities between vernix and SC, as well as the dynamic, variable nature of SC ceramide composition over time.

The fatty acid profile of vernix lipids includes branched-chain fatty acids (BCFA), a species that is not present in epidermal barrier lipids, as well as saturated, mono-unsaturated, and poly-unsaturated fatty acids (Table 1). Additionally, the fatty acid distribution in vernix lipids differed significantly by GA. Premature infants (29–36 wks GA) had significantly higher saturated and poly-unsaturated FAs and lower BCFA and mono-unsaturated FAs than full-term infants (≥37 wks GA) (Li et al., 2021).

**TABLE 1:** Profile of the fatty acid classes in vernix caseosa from premature and full-term infants (Li et al., 2021).

Species	Premature infants (% weight)	Full-term infants (% weight)
Saturated fatty acids	61.2 ± 1.22	35.8 ± 3.23
Monounsaturated fatty acids	3.0 ± 0.31	16.6 ± 5.09
Polyunsaturated fatty acids	10.5 ± 0.42	5.5 ± 0.02
Branched-chain fatty acids	25.3 ± 0.51	43.0 ± 1.87

Understanding the composition of vernix, which the infant ingests before birth, could have important pathophysiological consequences. For example, 20–30% of premature infants are affected by necrotizing enterocolitis, a potentially fatal intestinal inflammatory condition. In an animal model, dietary supplementation with 20% of vernix-type BCFAs reduced necrotizing enterocolitis by 50%, increased the intestinal microbiota diversity, and increased IL-10 three-fold versus the control whose diet was lacking in BCFA, indicating a protective role for this fatty acid in newborn intestines (Ran-Ressler et al., 2011). Additionally, data from an *in vitro* model demonstrated that induction of inflammation in intestinal cells with lipopolysaccharide (LPS) was associated with, a 20% reduction in cell viability was observed (Yan et al., 2018). However, when cells were treated with either vernix monoacylglycerides or vernix free fatty acids, the cell viability was restored. This study showed that the intestinal cells assimilated the BCFAs after treatment with vernix lipids. A putative role in prevention was suggested by experiments in which cells that were pretreated with vernix monoacylglycerides or vernix free fatty acids, followed by LPS exposure, expressed lower levels of IL-8 and NF-kB, suggesting that pretreatment with BCFA attenuated LPS-induced inflammation.

How epidermal lipids might mediate skin inflammation and immune function is unknown, but the mechanisms could include keratinocyte production of antimicrobial compounds, fibroblast migration, regulation of the rate of wound healing, and/or regulation of dendritic cells, for example, antigen uptake and activation of T cells (Kendall and Nicolaou, 2013). The impact of gestational age and gender has been studied with vernix samples from 156 infants in 3 GA categories, that is, 36–38 weeks, 39–40 weeks, and 41–42 weeks, revealing 54 lipid mediators (coefficient of variation <30% and in >70% of samples (Checa et al., 2015). Three classes of lipids were identified, namely, sphingolipids (*n* = 23), oxylipins (*n* = 43) and endocannabinoids (*n* = 14), and gender differences were noted (Checa et al., 2015). Sphingolipids are of interest for their potential role in skin barrier integrity and function, particularly to facilitate skin maturation and immunity in very premature infants (i.e., <28 weeks GA) who lack exposure to vernix. Within the sphingolipids, sphingomyelins increased with gestational age. The ceramide/sphingomyelin ratio (corrected for gender and maternal lifestyle) was significantly higher with increasing gestational age for chain lengths 12:0, 16:0, 18:0, 18:1, 24:0, and 24:1.

It is noteworthy that vernix from healthy full-term infants contained cytokines TNFα, IL8, IL1α, IL1β, IL6, MCP1, and IP10 (Narendran et al., 2010). The levels were substantially lower than in skin surface (stratum corneum) samples from premature infants, full-term infants, and adults (Narendran et al., 2010). This finding is consistent with the reduction of IL-8 and NF-kB in LPS-mediated intestinal cells (Yan et al., 2018). IL1α from the vernix covering may accelerate SC barrier maturation after birth (Jiang et al., 2009).

### 4.3 Vernix Lipids, Inflammation, and Filaggrin

Qiao et al. investigated the effects of vernix lipids (*n* = 10 infants) on the expression of a critically important skin protein, filaggrin (FLG), and markers of inflammation in normal human epidermal keratinocytes *in vitro* (Qiao et al., 2019). Inflammation, evidenced by increased amounts of cytokines tumor necrosis factor alpha (TNFα) and thymic stromal lymphopoietin (TSLP) was provoked by exposure of keratinocytes to polyinosinic:polycytidylic acid (poly I:C), a synthetic double-stranded RNA. This resulted in a dose-dependent reduction in cell viability (Qiao et al., 2019). Exposure to vernix lipids attenuated TNFα and TSLP levels, further supporting the antiinflammatory potential of vernix. In this work, keratinocytes treated with Poly I:C decreased FLG expression while the addition of 25, 50, and 100 μg/ml of vernix lipids increased filaggrin (FLG) expression relative to cells that were not treated with Poly I:C. (Qiao et al., 2019). In contrast, FLG expression decreased in keratinocytes that were treated with poly I:C. This work may have broader relevance to newborn infants as FLG is a precursor of the Natural Moisturizing Factor. FLG mutations are implicated in atopic dermatitis. Further investigation of the effect of vernix on atopic dermatitis is warranted.

### 4.4 Vernix Proteins

**FIGURE 2:**
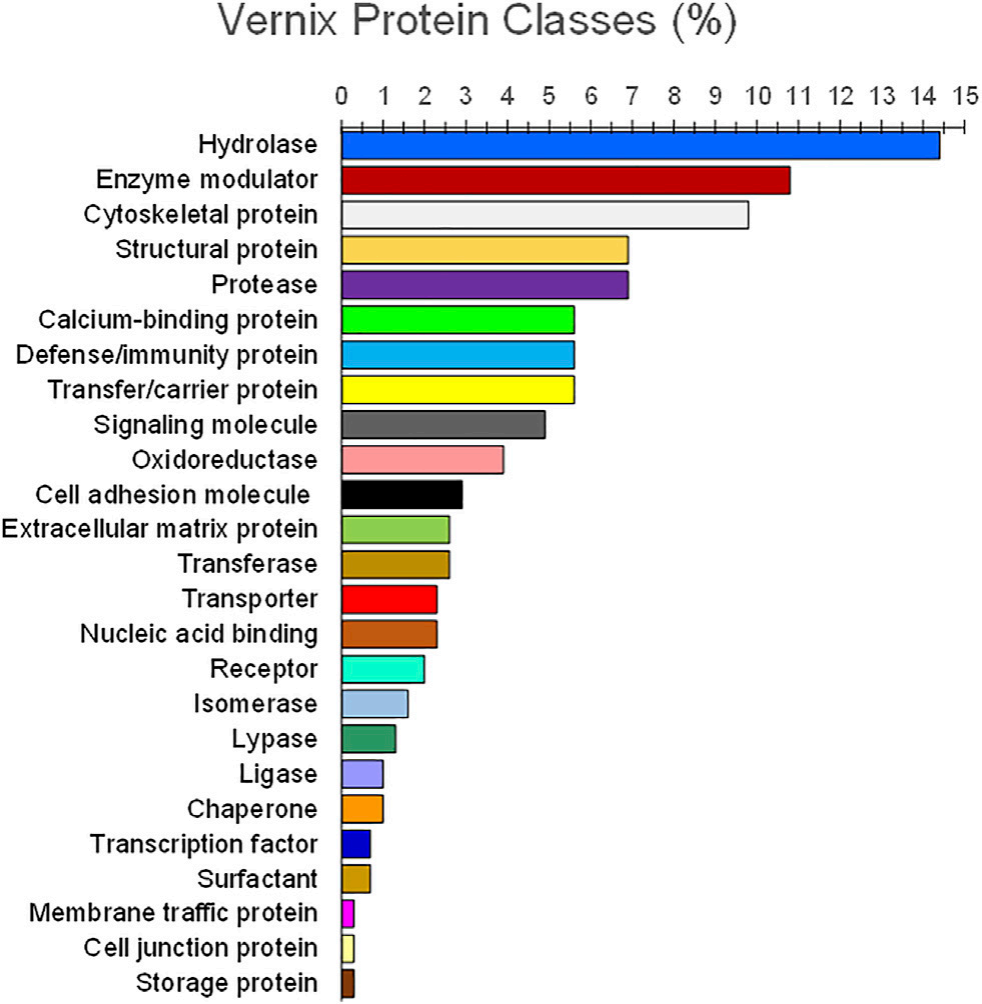
Functional classes of vernix proteins. Vernix was extracted and digested with trypsin, quantified by liquid chromatography–tandem mass spectrometry, and analyzed against the Swiss-Prot protein database (Holm et al., 2014). Proteins (*n* = 203) for *p* < 0.05 and belonging to 25 functional classes were identified (percent by weight). Hydrolases, proteases, and enzyme modulators encompassed 29, 22, and 22 proteins, respectively, with 11 proteins classified as immunity/defense.

Analysis of vernix proteins with 2D gel electrophoresis identified 41 proteins, including 16 associated with innate immunity (Tollin et al., 2006). They were: UBB (ubiquitin), S100A8, S100A9, S100A7, LYZ, NGAL, H2AC11, H2BC1, RNASE7, SLPI, CAMP (LL-37), MUC7, BPIFA1, PSMB2, ARG1, and SOD1. Additionally, the first 12 genes (UBB to MUC7) demonstrated antimicrobial properties (Tollin et al., 2006). Vernix contains antimicrobial proteins lysozyme and lactoferrin, localized in “granules” that may facilitate “quick release” in the presence of infectious agents (Akinbi et al., 2004). Vernix decreased specific perinatal pathogens, namely group B *Streptococcus*, K *pneumoniae,* and L *monocytogenes* (Akinbi et al., 2004). Holm, et al., analyzed 34 individual vernix samples using liquid chromatography tandem mass spectrometry and identified 203 proteins (Holm et al., 2014). Their analysis with multivariate and classification methods revealed 25 functional classes. Hydrolases, proteases, and enzyme modulators encompassed 29, 22, and 22 proteins, respectively, with 11 proteins classified as immunity/defense and generally consistent with Tollin et al., (2006); Holm et al., (2014). The 34 vernix samples were from 16 infants who had developed atopic dermatitis by 2 years of age and 18 non-atopic healthy controls. A comparison of the proteomic data found significantly reduced levels of both UBC (polyubiquitin-C) and CALM5 (calmodulin-like protein 5) in vernix of infants who later developed atopic eczema versus vernix from infants who did not develop eczema, shown in Figure 2 (Holm et al., 2014). Furthermore, investigation to determine whether these biomarkers are early indicators of atopic disease is clearly warranted, given the increased incidence and morbidity associated with this condition.

### 4.5 Vernix as a Skin Protectant

**FIGURE 3:**
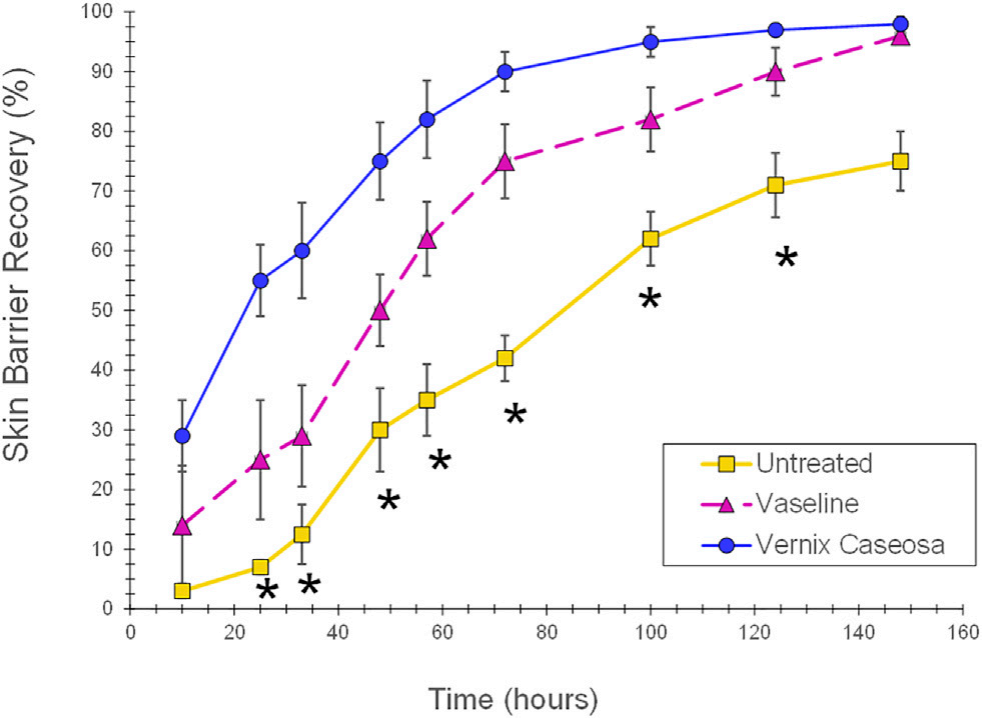
Skin barrier repair following application of vernix or petrolatum versus untreated skin. Skin barrier damage was created by repeatedly tape stripping the SC in the hairless mouse model. The damaged skin sites were treated with 5 mg/cm^2^ of vernix, 5 mg/cm^2^ petrolatum, or left untreated as controls and barrier recovery monitored over time (one-way ANOVA, posthoc Bonferroni correction, and *p* < 0.05) (Oudshoorn et al., 2009b). Vernix treated skin demonstrated a significantly increased rate of SC barrier repair compared to untreated, damaged control skin. In the same study, treatment of damaged skin with petrolatum also significantly increased the SC barrier repair rate versus the untreated control, but the skin was more erythematous and thickened compared to the vernix treated skin (*p* < 0.05). *Indicates significant difference for untreated skin versus vernix and petrolatum treated sites (*p* < 0.05).

Vernix has demonstrated multiple “protective” functions. Evidence of these actions includes the following. 1) Vernix was spread on a highly permeable fiber substrate to create films of known thicknesses *in vitro*. The vernix films impeded exogenous chymotrypsin transport and maintained the native enzyme activity that is necessary for epidermal development (Tansirikongkol et al., 2007b). 2) Normal adult skin was treated with native vernix and common skin creams petrolatum, Aquaphor and Eucerin, and an untreated control. Vernix-treated skin had a significantly higher peak water sorption value than all the cream treatments and the control, indicating that it binds exogenous water to the skin (Bautista et al., 2000). 3) In parallel cohorts of full-term infants, vernix was retained on the skin of one group and removed from the other group. The skin covered with vernix was significantly more hydrated, less erythematous, and had a lower surface pH than skin where the vernix was removed (Visscher et al., 2005). These differences were observed immediately after birth and 24 h later. 4) The SC from the vernix retained and vernix removed groups was sampled 24 h after birth and analyzed for the free amino acid (FAA) component of the natural moisturizing factor. Free amino acid levels were significantly higher for infants with vernix retained versus those with vernix removed where FFAs were extremely low or undetectable (Visscher et al., 2011b). The FFA appeared to originate from the vernix that was retained on the skin after birth. That is, native vernix contained FFAs. 5) Skin barrier damage was created by repeatedly tape stripping the SC in the hairless mouse model. The damaged skin, treated with vernix, demonstrated a significantly increased rate of SC barrier repair compared to untreated, damaged control skin (Oudshoorn et al., 2009a). In the same study, treatment of damaged skin with petrolatum also significantly increased the SC barrier repair rate versus the untreated control, but the skin was more erythematous and thickened compared to the vernix treated skin (Figure 3). 6) Wounds that were produced with 25 microns of laser energy (animal model) showed an increased rate of barrier recovery after 2 days of treatment with either vernix or a petrolatum-based cream compared to a wounded, untreated control (Visscher et al., 2011a).

In summation, the literature suggests that vernix protects the infant throughout fetal development and at birth, supporting its role in the innate immune function of the epidermis. Vernix appears during the last 10 weeks of gestation. Consequently, premature infants, particularly those <29 weeks GA at birth, lack exposure to significant amounts of vernix, raising these questions. What is the effect of exposure to vernix caseosa during gestation on the development of the innate immune system? How might the presumed positive effects of vernix be implemented to facilitate innate immune system development in very premature infants?

## 5 Epidermal Barrier After Birth

### 5.1 Full-Term Infants

The dramatic transition from aqueous *in utero* conditions to a dry, gaseous environment at birth initiates changes in the skin that are required for the full-term infant to survive and thrive. Remarkably, the epidermal barrier is intact and functional, despite submersion in amniotic fluid. This is in marked contrast to skin maceration and SC lipid disruption with prolonged water exposure in older children and adults (Warner et al., 1999; Ogawa-Fuse et al., 2019). Within minutes after birth, full-term skin hydration changes and varies due to the presence of vernix, infant care practices, for example, exposure to radiant warming, and body site (Visscher et al., 1999; Visscher et al., 2005). Despite prolonged exposure to water during gestation, a rapid decrease in hydration occurs consistently within the first day, followed by an increase over the first 2 weeks and suggesting SC adaptation to the drier environment (Visscher et al., 1999; Visscher et al., 2000; Visscher et al., 2005; Fluhr et al., 2012). The low transepidermal water loss (TEWL) observed in full-term newborn skin indicated a well-functioning epidermal barrier (Hammarlund et al., 1979; Fluhr et al., 2012; Ludriksone et al., 2014). A rapid humidity decrease (hairless mice) lead to increased DNA synthesis, lower free amino acid levels, dry skin, and lower filaggrin immunoreactivity, due to decreased epidermal keratohyalin granules (Scott and Harding, 1986; Denda et al., 1998a; Visscher et al., 1999; Katagiri et al., 2003).

Full-term skin pH is nearly neutral at birth, decreases significantly by day 4 (Visscher et al., 2000) and then gradually continues to decrease over the next few months. An acidic skin pH is important in establishing the skin barrier as it promotes the effective functioning of enzymes required for SC development and integrity, that is, lipid metabolism, bilayer structure formation, ceramide synthesis, lipid bilayer formation, and desquamation (Rippke et al., 2002; Schmid-Wendtner and Korting, 2006). The skin pH reduction after birth is due to multiple mechanisms, including 1) filaggrin proteolysis to amino acids, pyrrolidone carboxylic acid, and urocanic acid; 2) secretory phospholipase hydrolysis to FFA; 3) acidification in the lower SC by a Na^+^H^+^ antiporter mechanism (NHE1); 4) melanin granule dispersion to release H+; and 5) cholesterol sulfate production of H^+^(Elias, 2017).

Full-term skin microbiota colonization begins at birth (Capone et al., 2011; Cuenca et al., 2013) and is populated by *Lactobacillus*, *Propionibacterium*, *Streptococcus,* and *Staphylococcus*, differing by body site at 6 weeks of life (Chu et al., 2017). Skin microbiota contributes to innate immunity by regulating antimicrobial peptides, including cathelicidins and β-defensins, and responding to inflammation via IL1α (Naik et al., 2012). *S. epidermidis* and *hominis* produce antimicrobial peptides that are noxious to *S. aureus* (Chen et al., 2018a; b). Skin bacteria and yeasts hydrolyze sebaceous gland triglycerides to glycerin and free fatty acids (Yosipovitch et al., 2000) that, in turn, have antimicrobial properties and contribute to skin surface acidity (Eyerich et al., 2018).

### 5.2 Premature Infants

The epidermal barrier is under-developed in premature infants at birth, particularly those <29 weeks GA, putting them at risk for infection and increased permeability to both internal water loss and external deleterious agents (Evans and Rutter, 1986; Cartlidge, 2000; Rutter, 2000). The skin is easily injured or torn due to deficiencies in dermal structural proteins (Eichenfield and Hardaway, 1999). Although epidermal barrier development is rapid upon exposure to a dry environment at birth (Harpin and Rutter, 1983; Okah et al., 1995; Agren et al., 2006), very premature stratum corneum is not fully competent at 1 month of life, as indicated by a considerably higher transepidermal water loss (TEWL) compared to full-term infants (Agren et al., 1998).

The preterm skin surface pH decreased following birth but the rate was slower for infants weighing less than 1,000 g than for infants weighing more than 1,000 g. The decrease was faster during postnatal week 1 versus weeks 2–4 (Fox et al., 1998). The interaction of GA and postnatal age significantly influenced the rate of pH reduction (Green et al., 1968).

Infection is one cause of premature birth. In utero exposure to infectious agents and/or to antibiotics before birth is likely to impact the microbiome soon after birth. Infants <32 weeks GA demonstrated a decrease in bacterial richness during postnatal weeks 1 and 2, followed by an increase. However, the bacterial diversity was lower in premature infants than it was in full-term infants (Pammi et al., 2017). Firmicutes and Proteobacteria were the most abundant phyla in premature infants. The implications of the skin microbiome and maternal antibiotic use are areas that warrant further investigation.

## 6 Epidermal Barrier Maturation- Proteomics

The risks of infection and skin damage in premature infants are considerable. Consequently, the facilitation of epidermal barrier maturation and immune function is a critically important aspect of clinical practice. A fundamental understanding of the biological processes governing skin maturation will enable the implementation of effective skincare practices, for example, humidification, topical treatments, and implementation of appropriate antisepsis measures.

The relatively recent emergence of quantitative, noninvasive analytical methods has enabled simultaneous measurements of protein and non-protein biomarkers of epidermal barrier status and immune function. A highly specific, quantitative analysis of the outer stratum corneum via noninvasive collection techniques revealed important differences in innate immune biomarkers in premature infants ≤32 weeks GA compared to full-term infant and adult samples. Proinflammatory cytokines IL1β, IL6, MCP1, and IL8 and structural proteins involucrin and albumin were significantly higher in premature infants (*p* < 0.05), and involucrin and albumin levels were inversely related to GA (Narendran et al., 2010). These initial findings prompted a more detailed investigation. Stratum corneum biomarkers of antimicrobial function and late cornification were hypothesized to be lower in premature infants than in full-term infants and later normalize over 3–4 months after birth.

Subsequently, targeted proteomic analysis of skin surface (stratum corneum) biomarkers and established biophysical measures of barrier function were used to determine changes over time. The cohorts included 61 newborn infants grouped by GA, specifically: premature <34 wks GA (PT), late premature ≥34-< 37 wks GA (LPT), and full-term ≥ 37 wks GA (FT) (Visscher et al., 2020). Infant biomarkers were compared to adult values (i.e., a widely studied, established steady-state condition subject parents, *n* = 34) at two-time points, 4–8 days after birth, and 2–3 months later when the three infant groups were at comparable gestational ages of 46–48 weeks.

**FIGURE 4:**
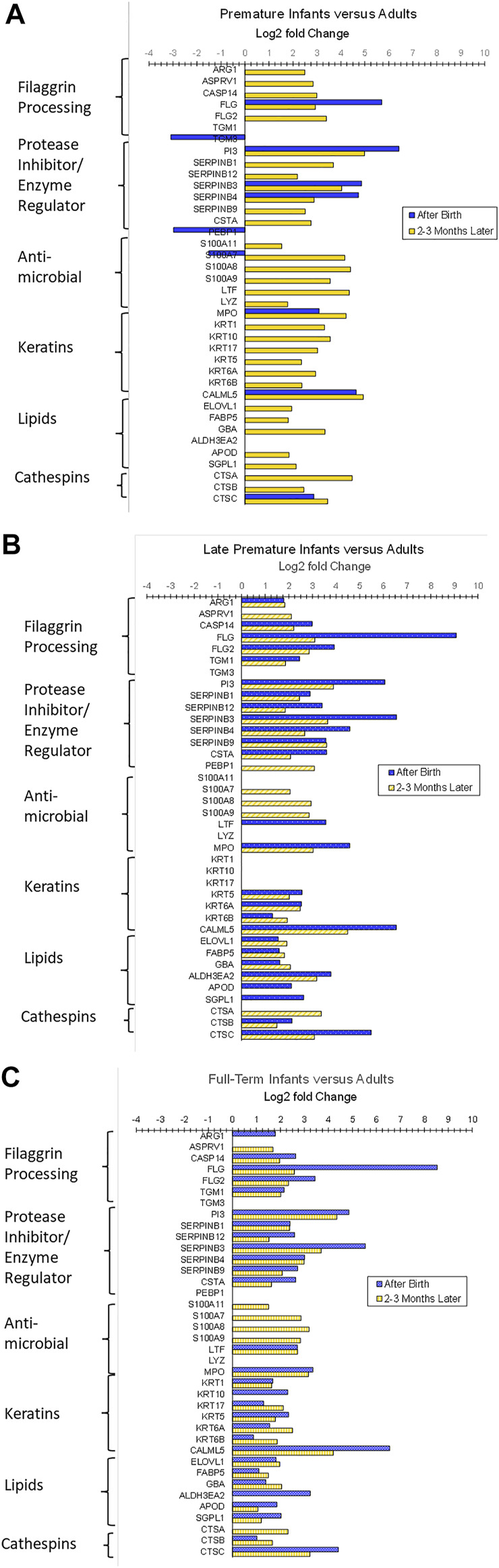
Differentially expressed biomarkers for infants 4–8 days after birth and 2–3 months later at corrected GAs of 46–48 weeks versus adult skin. Two sequential skin surface samples (stratum corneum) were collected from the lower legs of 61 infants at each time and from the volar forearms of 34 adults (parent) at one time. Samples were extracted, quantified with liquid chromatography tandem mass spectrometry, and analyzed with targeted proteomics (*p* < 0.05) (Visscher et al., 2020). The proteins classified by function were: filaggrin processing, protease inhibitor/enzyme regulators, antimicrobials, keratins/structural proteins, lipid processing, and cathepsins (*p* < 0.05). **(A–C)** show the log2 fold changes for the specific proteins in each class versus adults for PT, LPT, and FT infants at both times. The differentially expressed biomarkers were decidedly different for infant skin compared to stable adult skin. For PT infants, the differentially expressed proteins increased from 12 to 54 versus adults over 2–3 months, suggesting substantial adaptive changes over time.

The sets of differentially expressed biomarkers at both time points were decidedly different than those in stable adult skin. After birth, the expression of 40 biomarkers in FT, 38 in LPT, and 12 in PT was higher compared to adults (*p* < 0.05). Two-three months later, the expression of 40 biomarkers in FT, 38 in LPT, and 54 in PT was higher versus adults (*p* < 0.05). The differentially expressed proteins classified by function were: filaggrin processing, protease inhibitor/enzyme regulators, antimicrobials, keratins/structural proteins, lipid processing, and cathepsins (*p* < 0.05). Figures 4A–C show the log2 fold changes for specific proteins by class for PT, LPT, and FT infants versus adults at both times. The number of differentially expressed proteins increased from 12 to 54 for PT infants versus adults over the 2–3 month time period, suggesting substantial adaptive changes over time.

**FIGURE 5:**
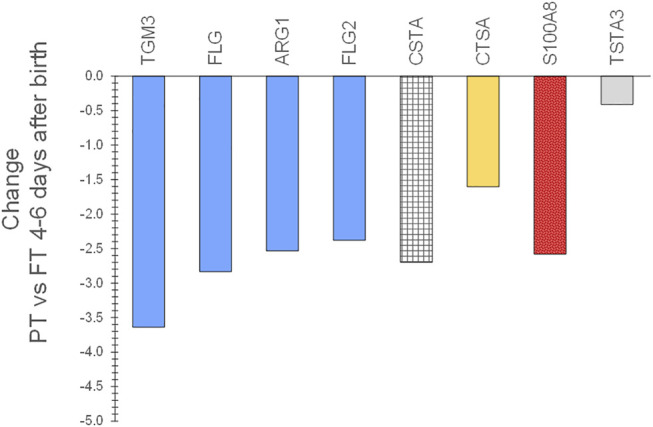
Changes in SC proteins for PT infants compared to FT infants 4–8 days after birth. Two sequential skin surface samples (stratum corneum) were collected from the lower legs of 61 infants at each time and from the volar forearms of 34 adults (parent) at one time. Samples were extracted, quantified with liquid chromatography tandem mass spectrometry, and analyzed using targeted proteomics (*p* < 0.05) (Visscher et al., 2020). PT infant SC had decreased expression of filaggrin processing biomarkers FLG, FLG2, AGR1, and TGM3, antimicrobial S100A8, protease inhibitor CSTA, and protective protein CTSA (cathepsin A) soon after birth compared to FT infant SC.

**FIGURE 6:**
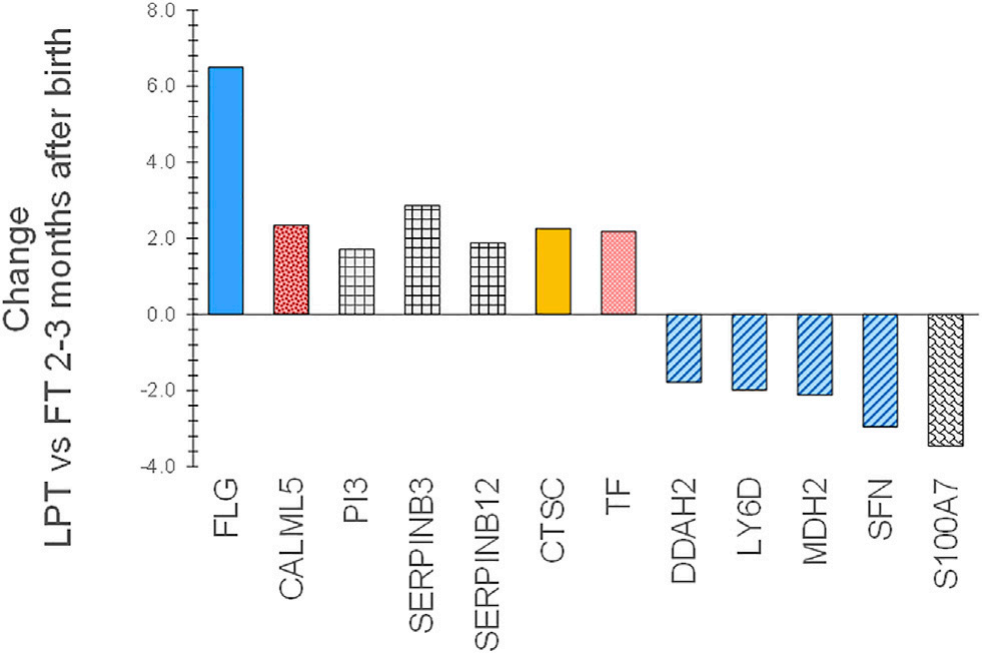
Changes in SC proteins for LPT infants compared to FT infants 2–3 months after birth at comparable corrected GA. Two sequential skin surface samples (stratum corneum) were collected from the lower legs of 61 infants at each time and from the volar forearms of 34 adults (parent) at one time. Samples were extracted, quantified with liquid chromatography tandem mass spectrometry, and analyzed using targeted proteomics (*p* < 0.05) (Visscher et al., 2020; Visscher et al., 2021). LPT infants had increased expression of protease inhibitors PI3, SERPINB3, and SERPINB12, as well as FLG, CALML5, CTSC, and TF versus FT infants. Expression of S100A7, LY6D, SFN, MDH2, and DDAH2 was lower in LPT compared to FT 2–3 months later. These findings suggest that the rate of change of specific aspects of epidermal barrier development may vary with GA and/or time from birth.

Expression patterns of SC biomarkers between the infant groups were examined to gain insight into the effects of gestational age at birth and after 2–3 months of life. PT infant SC had decreased expression of filaggrin processing biomarkers FLG, FLG2, AGR1, and TGM3, antimicrobial S100A8, protease inhibitor CSTA, and protective protein CTSA (cathepsin A) soon after birth compared to FT infant SC (Figure 5). The protein expression did not differ for PT versus FT 2–3 months later. LPT and FT infants had comparable protein expression soon after birth but LPT had increased expression of protease inhibitors PI3, SERPINB3, and SERPINB12, as well as FLG, CALML5, CTSC, and TF (Figure 6). Expression of S100A7 (antimicrobial), LY6D, SFN, MDH2, and DDAH2 was lower in LPT compared to FT at similar corrected GA 2–3 months later. These findings suggest that the rate of change of specific aspects of epidermal barrier development may vary with GA and/or time from birth.

### 6.1 Epidermal Immunity

Expression patterns for biomarkers of innate immunity by GA and over time warrant further comment. Soon after birth, increased expression of the antimicrobial proteins MPO (all infants) and LTF (LPT, FT) were noted in adults. Two to three months later, the expression of biomarkers S100A8, S100A9, S100A7, and S100A11, as well as MPO, LTF, and LYZ (PTs), had increased significantly versus adults (Figures 4A–C). S100A7, S100A8, and S100A9 prompt keratinocytes to produce cytokines and chemokines. In turn, cytokines can promote the production of S100A7, S100A8, and S100A9, and, thereby, respond to threats (stressors) to facilitate immunity against pathogens (Lee et al., 2012; Lesniak and Graczyk-Jarzynka, 2015). Increased expression of S100A7, S100A8, S100A9, and S100A12 occur in inflammatory skin conditions with epidermal barrier defects, that is, atopic dermatitis and psoriasis (Oestreicher et al., 2001; Sugiura et al., 2005; Benoit et al., 2006; Wolk et al., 2006). Two to three months after birth, several clinical measures of barrier status demonstrated higher TEWL (FT) versus adults, higher visual dryness (PT, FT) and lower SC cohesion (PT, FT) (Visscher et al., 2020). Consequently, the increased S100 protein expression levels may occur in response to multiple factors, including pathogen exposure and minor barrier injury.

The increased expression of filaggrin processing biomarkers FLG, FLG2, ASPRV1, CASP14, and TGM1 for all infants compared to adults 2–3 months after birth was associated with changes in the products of filaggrin proteolysis, that is, natural moisturizing factor (NMF), histidine, proline, urocanic acid, and pyrrolidone carboxylic acid (PCA) that were quantified with reverse phase high-performance liquid chromatography and tandem mass spectrometry (Wei et al., 2016). NMF, PCA, histidine, and proline amounts were significantly higher for every infant group versus adults 2–3 months later (*p* < 0.05). In contrast, after birth, NMF, PCA, histidine, and proline levels were lower for all three infant groups versus adult samples (*p* < 0.05). The NMF increase was associated with a skin surface pH reduction for all infant groups (data not shown), and this acidification of the epidermal barrier processes is necessary to provide immunity via the promotion of colonization with effective microbiota.

All three infant groups (PT, LPT, FT) had higher levels of 9 biomarkers versus adults shortly after birth (FLG, SERPINB3, SERPINB4, PI3, MPO, CALML5, CTSC, ALB, TF, Figures 4A–C), likely indicating their importance in newborn skin development and maturation. The functions and possible implications of these proteins are discussed below. Increased FLG was associated with reduced NMF in infants, potentially due to inhibition of FLG proteolysis at high humidity described *in utero* (Scott and Harding, 1986). SERPINB3 and SERPINB4 are protease (e.g., serine, cysteine) inhibitors, including proteases generated by infectious pathogens (Sun et al., 2017). PI3 inhibited keratinocyte desquamation prior to terminal differentiation (Nakane et al., 2002) and kallikrein proteolysis (McGovern et al., 2017) and is a component of the corneocyte envelope (Steinert and Marekov, 1995), functions that are important to the provision of the physical epidermal barrier. While identified in the SC, MPO was produced in immune cells, for example, neutrophils, and lymphocytes (Khan A. A. et al., 2014; Liu et al., 2015), was higher in infected wounds (Gabr and Alghadir, 2019), and was higher under conditions of oxidative stress (Khan et al., 2018) and inflammation (Voss et al., 2018). Higher CALML5 levels were implicated in terminal differentiation (Sun et al., 2015) and barrier repair (atopic dermatitis) (Donovan et al., 2013). CTSC prompted serine protease generation in immune cells (Meyer-Hoffert, 2009). High ALB was associated with reduced skin hydration, consistent with our observation of lower hydration/skin dryness, particularly in LPT and FT shortly after birth. TF was associated with inflammation (Mehul et al., 2017).

Over the time from birth until 2–3 months later, a greater number of biomarkers were differentially expressed for infants versus adults, in addition to those involving filaggrin processing. S100A7, S100A8, S100A9, and MPO were significantly higher for all infants versus adults (Figures 4A–C). Protease inhibitors/enzyme regulators, PI3, SERPINB3, and SERPINB4 remained higher for all infant groups versus adults, and SERPINB1, SERPINB9, SERPINB12 and CSTA became significantly higher over time (Figures 4A–C). SERPINB1, located in macrophages and the cytoplasm and granules of neutrophils (Majewski et al., 2016), functions as an antimicrobial in infections and can guard against apoptosis. SERBINB9 has been described to react with enzymes in bacteria, yeasts, and fungi (Meyer-Hoffert, 2009) and serves in host-defense against bacteria and viruses in the lung, another epithelial tissue (Askew and Silverman, 2008). SERPINB12 is ubiquitous in human tissue, including the epidermis and eccrine duct, and is thought to guard macrophages from their internal protease inhibitors as well as from exogenous sources (Niehaus et al., 2015).

Collectively, these unique protein expression profiles suggest that the processes and pathways regulated by these proteins continue to be important for the provision of epidermal immunity well after birth. Neonates respond to multiple system transitions at birth by 1) producing NMF and lowering skin pH, 2) mitigating desquamation by inhibiting specific protease activity, and 3) increasing the antimicrobial features of the epidermal barrier. Neonatal skin must adapt over time to provide a sufficient level of epidermal immunity and maturation.

## 7 Infant Skin Genomics

Neonatal epidermal development and immunity were further examined via genomic analysis of full-thickness skin samples from newborns who required surgery. The hypothesis was that infant skin would exhibit increased expression of innate immunity genes and adult skin would have increased expression of epidermal barrier genes. Genomic analysis of newborn infant skin (*n* = 27) was compared to ultraviolet radiation protected adult skin (age 20–60 years, *n* = 43) to differentiate the physiological and structural features at the biological, molecular, and cellular levels as previously described (Visscher et al., 2021).

**FIGURE 7:**
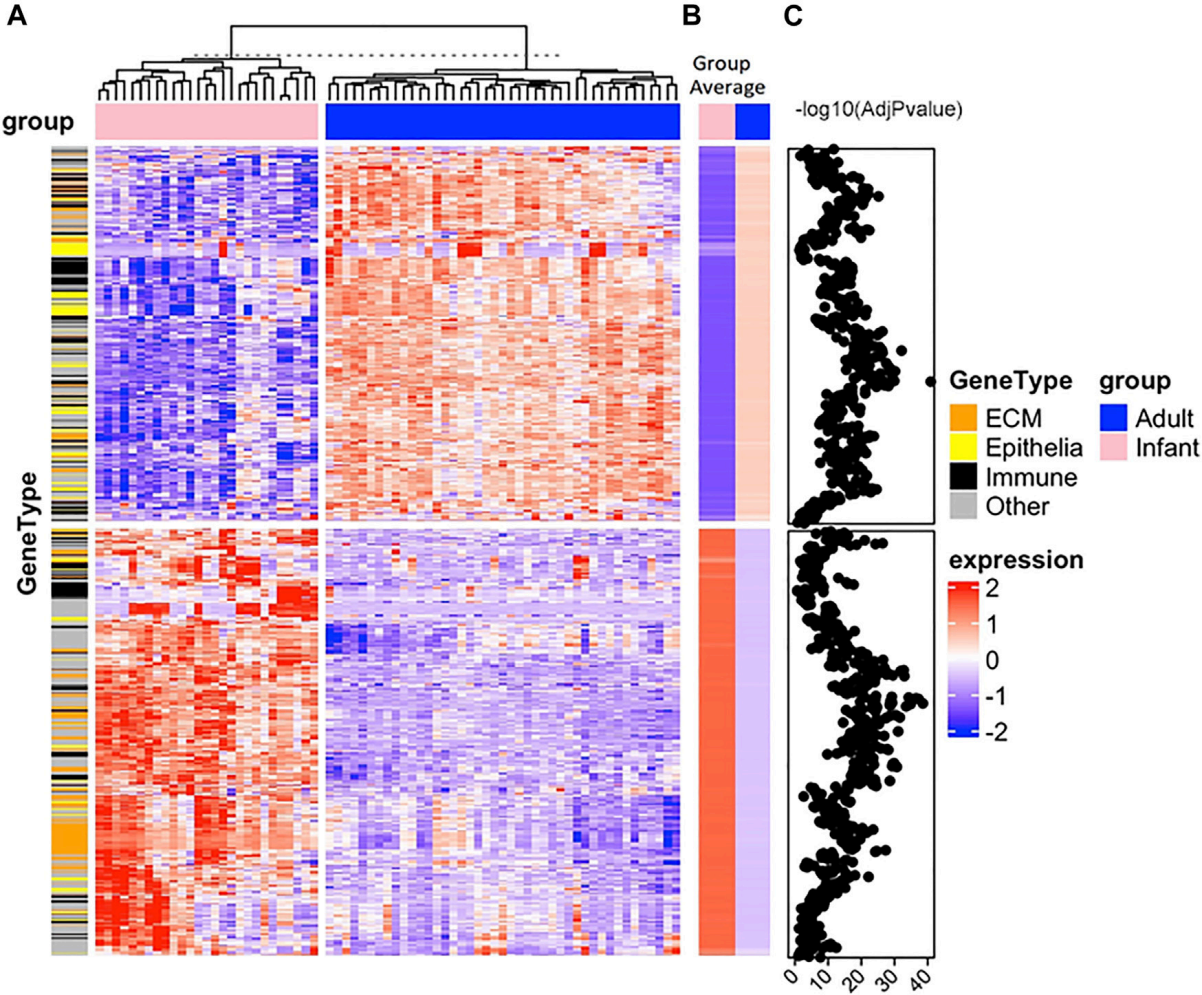
Hierarchical clustering analysis of differentially expressed genes in newborn infant and adult skin samples. Full-thickness tissue samples (body site, non-foreskin) from 27 infants were collected at non-elective surgery and buttocks tissue (protected from ultraviolet radiation exposure) from 43 adults was processed to collect total RNA (Visscher et al., 2021). Gene expression was determined from mRNA using Affymetrix GeneTitan U219 array plates. The lowest 30% of the 49,386 gene transcripts were removed, assayed for quality, data was normalized and Log2 transformed, analyzed using linear models and differential expression analyses and analyzed and analysis of variance with a term for the combination was conducted, as previously described (Merico et al., 2010; Supek et al., 2011; Yu et al., 2012; Gu et al., 2016; Szklarczyk et al., 2019; Visscher et al., 2021). Rigorous quality control was applied and all data were MIAME compliant. The Empirical Bayes method (limma R-package) was used to test comparisons. Test statistics were moderated with the Empirical Bayes method (limma R-package). The Benjamini–Hochberg correction was used to control for false discovery rates. The complete linkage method using the R hclust function was used to perform hierarchical clustering. Genes that were significantly expressed were analyzed for enrichment of biologic themes (Gene Ontology) using the clusterProfiler package (Yu et al., 2012), EnrichmentMap (Merico et al., 2010), g:profiler (Raudvere et al., 2019), Revigo (Supek et al., 2011) and String database (Szklarczyk et al., 2019). The transcriptomics is in the NCBI Gene Expression Omnibus (GEO) repository with the dataset accession number GSE181022. Panel **(A)** is a heatmap of the normalized expression values (based on z-score) of the 1,086 differentially regulated genes with adjusted *p* value <0.05 and absolute fold change ≥1.5 for the two groups, infants (pink) and adults (blue). Euclidean distances between each sample were determined and cluster analysis was performed with an unsupervised hclust algorithm. Samples formed two clusters. From a hierarchical cluster, analysis genes were grouped for similarity where each column is an individual sample and each row is a single gene. Extracellular matrix (ECM, orange), immune-related (black), and epithelial (yellow) genes are indicated on the annotation bar under the gene type. Panel **(B)** is a heatmap of group average values for infants (pink) and adults (blue). Panel **(C)** are the values of the negative log_10_ of the adjusted *p* values from the Limma testing for adults versus infants for each gene. The z-scores are shown in the blue-white-red gradient where 2 is the darkest red color, 0 is white and -2 is the darkest blue color shown on the right and labeled as “expression”. Many negative log_10_ adjusted *p* values were greater than 10, indicating large differences.

There were numerous differences across biological processes, with 1,086 probes differentially expressed in infant skin versus adult skin with 508 probes increasing while 578 probes decreased. Hierarchical clustering analysis of the probe normalized expression values (|FC| ≥ 1.5, adjusted *p* value <0.05) was performed. Limma testing (negative log_10_ (AdjPvalue)) showed many values over 10 for infants versus adults, representing large differences (Figure 7).

**FIGURE 8:**
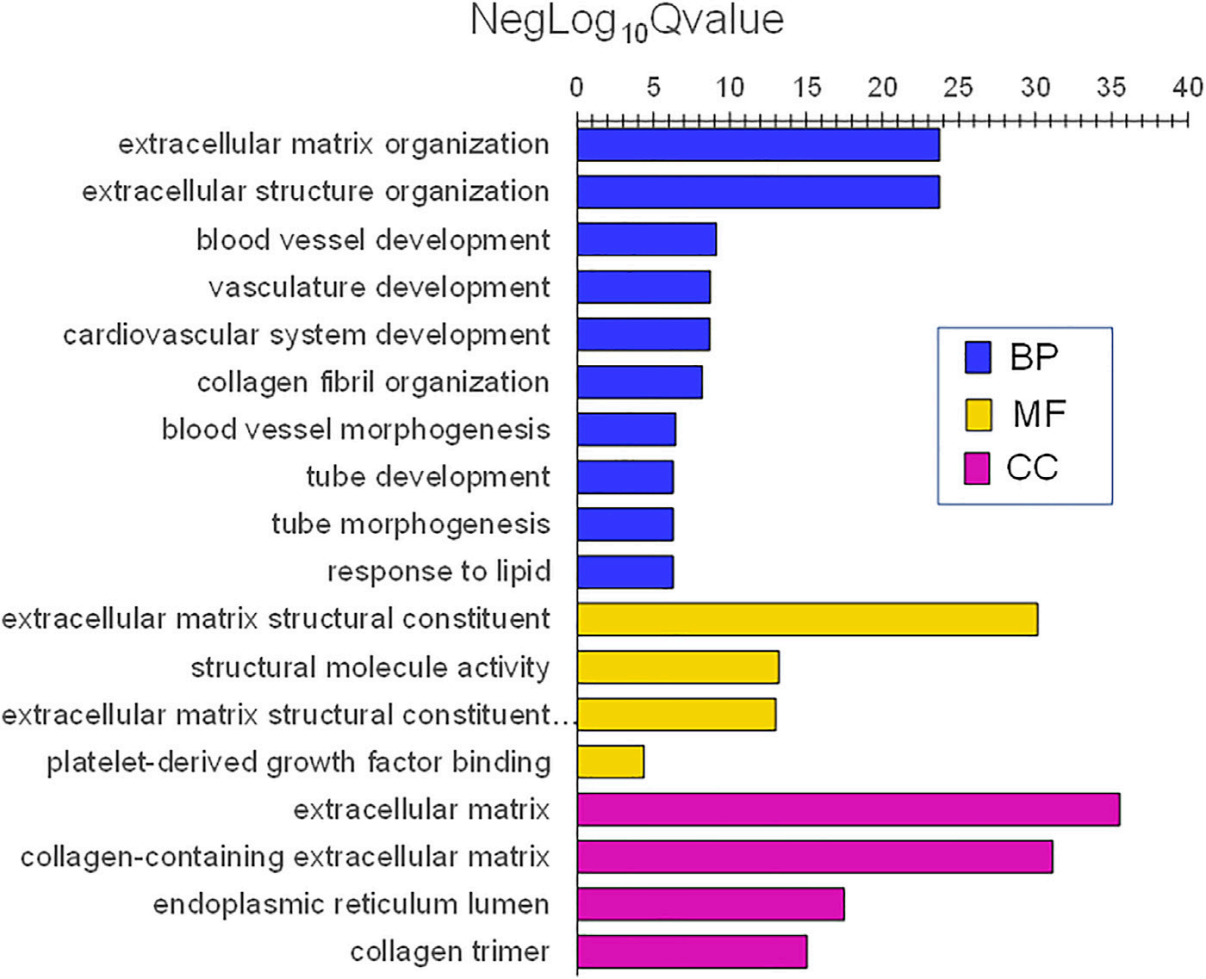
Gene ontology themes with enriched gene expression in infant skin. Significantly expressed genes were analyzed for enrichment of biologic themes (Gene Ontology) using the clusterProfiler package (Yu et al., 2012), EnrichmentMap (Merico et al., 2010), and g:profiler (Raudvere et al., 2019). Significant pathways were selected using the false discovery rate (FDR)adjusted *p* values. NegLog_10_Qvalue indicates -Log_10_FDR adjusted *p* value. The lowest adjusted *p* values (highest NegLog_10_Qvalue) were biological processes (BP) extracellular matrix (ECM) organization and ECM structure organization. Others included system development (e.g., blood vessel, cardiovascular, tube), and response (e.g., to lipid). The most significant molecular functions (MF) were ECM matrix structural constituent and structural molecule activity. ECM and collagen-containing ECM were the most significant cell components (CC).

Infant skin was enriched in genes implicated in many gene ontology (GO) themes for biological processes, molecular functions, and cell components. The lowest adjusted *p* values (highest NegLog_10_Qvalue) as categorized by GO biological processes (BP) were extracellular matrix (ECM) organization and ECM structure organization. Others included system development (e.g., blood vessel, cardiovascular, tube) and response (e.g., to lipid) (Figure 8). The most significant GO molecular functions (MF) were ECM matrix structural constituent and structural molecule activity. GO cell components (CC) with the lowest adjusted *p* values were ECM and collagen-containing ECM.

**FIGURE 9:**
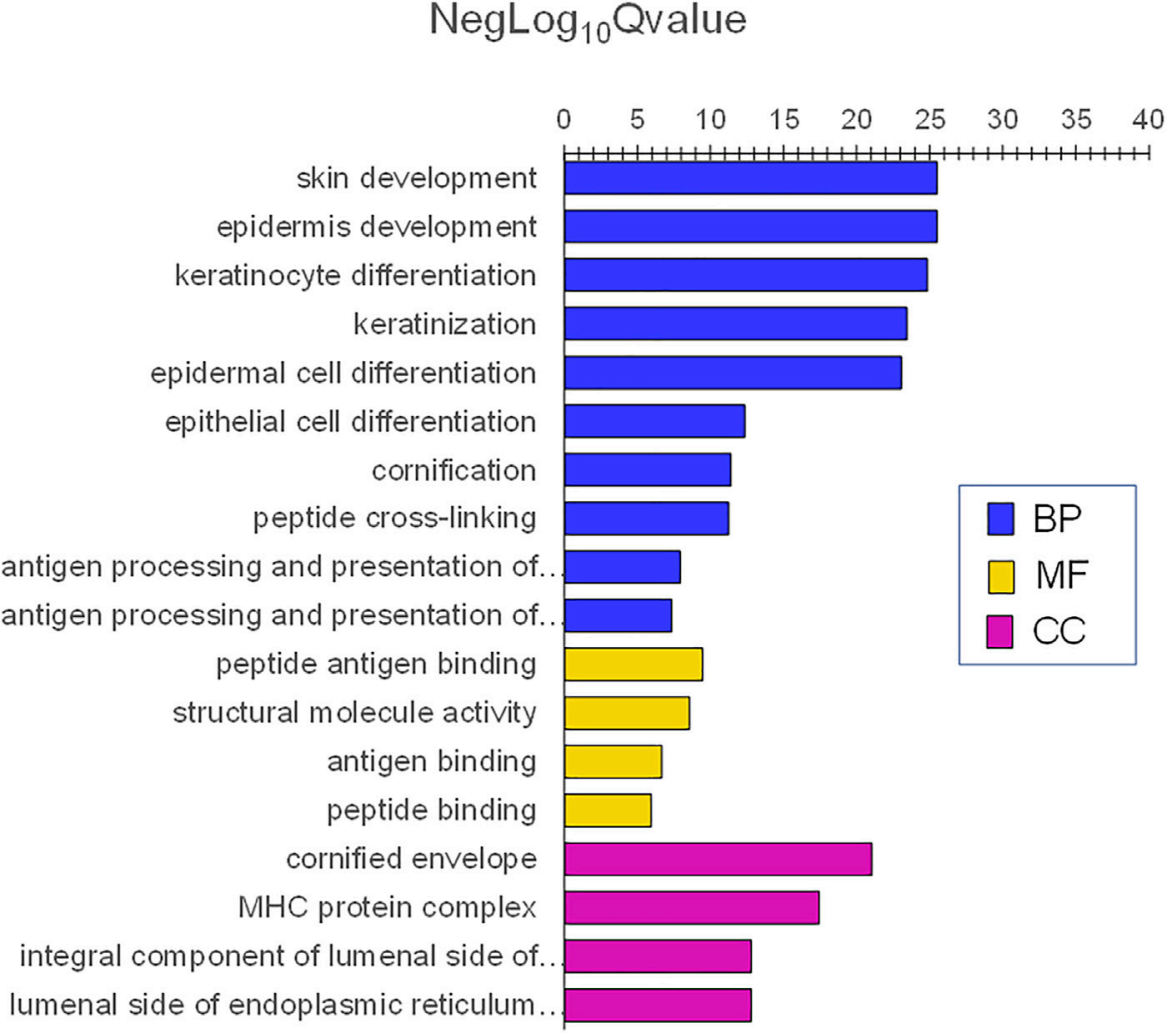
Gene ontology themes with enriched gene expression in adult skin. Significantly expressed genes were analyzed for enrichment of biologic themes (Gene Ontology) using the clusterProfiler package (Yu et al., 2012), EnrichmentMap (Merico et al., 2010), and g:profiler (Raudvere et al., 2019). Significant pathways were selected using the false discovery rate (FDR)adjusted *p* values. NegLog_10_Qvalue indicates -Log_10_FDR adjusted *p* value. The lowest adjusted *p* values for BP were skin development, epidermis development, keratinocyte differentiation, keratinization, and cornification. Immune BPs included antigen processing and presentation of exogenous antigen, major histocompatibility protein complex, and antigen-binding were also prominent. Highly significant MF was peptide antigen binding and structural molecule activity and CC was a cornified envelope and major histocompatibility complex (MHC) protein complex.

In contrast, adult skin was enriched in genes involved with skin and epidermis development. The lowest adjP values for BPs were skin development, epidermis development, keratinocyte differentiation, keratinization, and cornification. Additionally, immune BPs including antigen processing and presentation of exogenous antigen, major histocompatibility protein complex, and antigen-binding were also prominent (Figure 9). Highly significant MF were peptide antigen binding and structural molecule activity and those for CC were cornified envelope and major histocompatibility complex (MHC) protein complex (Figure 9). Complete lists of the infant and adult processes for adjusted *p* values <0.0001 are listed in Supplementary Tables S1 and S2.

Kyoto Encyclopedia of Genes and Genomes (KEGG) analysis revealed 15 enriched pathways in infant tissues wherein protein digestion and absorption, PI3K-Akt signaling pathway, human papillomavirus infection, ECM receptor interaction, and focal adhesion had the lowest adjusted *p* values. Adult tissues had 38 enriched KEGG pathways, including those associated with *Staphylococcus aureus* infection, allograft rejection, immune disease, infectious disease, cancer, transport and catabolism, endocrine or endocrine disease and antigen processing and presentation had the lowest adjusted *p* values.

Analysis of data using REACTOME revealed 20 pathways with the genes differentially expressed in infant tissues, with adjusted *p* values <0.001 compared with adults (Table 2). Extracellular matrix organization, a top-level pathway, had the lowest adjusted *p* value. Also enriched in infants were sub-pathways degradation of the extracellular matrix, ECM proteoglycans, collagen formation, integrin cell surface, non-integrin membrane-ECM interactions, and laminin interaction. REACTOME analysis showed 5 pathways with genes differentially expressed in adult tissues, with adjusted *p* values <0.001 compared with infants (Table 2). The pathways included keratinization and formulation of the cornified envelope, that is, epidermal barrier, as well as interferon gamma signaling and endosomal/vacuolar pathway, that is, immune function.

**TABLE 2:** REACTOME pathways with the genes differentially expressed in infant tissues with adjusted *p* values <0.001 compared with adults and for adult tissues with adjusted *p* values <0.001 compared with infants.

Increased in infants	*p* value	Increased in adults	*p* value
Extracellular matrix organization	1.00E-28	Keratinization	1.01E-23
Degradation of the extracellular matrix	5.62E-19	Formation of the cornified envelope	2.36E-23
ECM proteoglycans	1.51E-17	Developmental Biology	1.79E-08
Collagen formation	9.23E-16	Interferon gamma signaling	4.00E-06
Collagen biosynthesis and modifying enzymes	2.54E-13	Endosomal/Vacuolar pathway	8.29E-06
Assembly of collagen fibrils and other multimeric structures	6.27E-13		
Collagen chain trimerization	1.35E-12		
Collagen degradation	3.86E-11		
Regulation of Insulin-like Growth Factor (IGF) transport and uptake by Insulin-like Growth Factor Binding Proteins (IGFBPs)	2.12E-10		
Integrin cell surface interactions	3.53E-09		
Post-translational protein phosphorylation	1.59E-07		
Crosslinking of collagen fibrils	3.76E-07		
Non-integrin membrane-ECM interactions	5.64E-07		
MET activates PTK2 signaling	1.9735E-06		
Laminin interactions	1.9735E-06		
NCAM1 interactions	3.2334E-06		
Metal sequestration by antimicrobial proteins	7.9462E-06		
MET promotes cell motility	3.1644E-05		
Signaling by PDGF	4.2549E-05		
Signaling by receptor tyrosine kinases	6.1897E-05		

Gene expression analyses were conducted on infants, adults, children, and adolescents to understand the progression of atopic dermatitis (AD) over time. Lesional and nonlesional tissue samples from individuals with AD were compared, with samples from non-AD infants, adults, children, and adolescents serving as normal controls (Renert-Yuval et al., 2021). The infant-adult control comparison revealed more differentially expressed genes than any other. Several immune genes were increased in infants, namely, S100A7, CTLA4, S100P, CXCR4, CCL4L1, CCL25, CCL4, CSCL2, IL6, IL10, CCL3, IL32, TNFRSP4, TNFB3 and CCL16 (criteria of the fold change of 2 and false discovery rate of <0.05) (Renert-Yuval et al., 2021). The following epidermal barrier genes were significantly increased in non-atopic adults versus infants: LCE1F, LCE2B, LCE2C, LC#2D, KRT2, SCEL, CLDN11, EREG, FLG, ELOVL5, FADS1, FADs2, CLDN1, FABP7, SCPP1, and CLDN8. In comparison, the immune genes S100A7, S100P, and CXL2 were significantly increased in newborn infants versus adults, and the epidermal barrier genes LCE1F, LCE2B, LCE2C, LCE2D, KRT2, and CLDN1 were significantly increased in adults compared to newborn infants in the previous study (Visscher et al., 2021). The infants in Renert-Yuval, et al., were somewhat older 14 ± 10 months (range 3–36 months) at tissue compared to 1.5 ± 2.6 months (range 0.1–11.3) in Visscher, et al. The age difference may account for the differences in gene expression. The TH17/TH22 genes IL20, IL22, S100A7, S100A9, S100A12, S100A8, CCL20, and PI3 were significantly increased in infants compared to the older groups in Renert-Yuval, et al. Likewise, the expression of S100A7, S100A8, S100A9 and PI3 were significantly increased in infants versus adults in Visscher, et al.

## 8 Discussion

Compared to adult skin, infant skin exhibited increased gene expression for extracellular matrix and development, among multiple processes. For adult skin, compared to infant skin, gene expression was higher for epidermal homeostasis and antigen processing/presentation, that is, adaptive immune function, and others. The adult epidermal barrier is constantly renewing while the infant barrier development is “in progress”. The newborn infant depends upon the innate immune system, including the extracellular matrix, to protect against microbiota and the relatively hostile environment after birth, while stimulating adaptive immunity. Table 3 provides an overview of the most important features of premature and full-term infants at birth and over the first few postnatal months relative to the benchmark of normal, healthy adult skin.

**TABLE 3:** Overview of the most important features of premature and full-term infants at birth and over the first few postnatal months relative to the benchmark of normal, healthy adult skin.

Skin feature	Premature infant	Full-term infant
Barrier integrity (TEWL, g/m^2^/hr)	Initially higher vs. FT and adult then decreases over time	Comparable to adults at birth
Hydration	Higher initially vs. FT and adult, decreases then increases	Initially lower vs. PT and adult, then increases
pH	Comparable to FT, higher vs. adult then decreases more slowly vs. FT	Comparable to PT, higher vs. adult, then decreases
Visual dryness/scaling	Lower vs. FT, comparable to adult then increases before decreasing	Higher vs. PT and adult initially, then decreases
Visual erythema	Higher vs. adult at birth and 2–3 months later	Higher vs. adult at birth and 2–3 months later
SC cohesion	Initially comparable to adults then decreasing before increasing again	Lower vs. PT and adult initially then increasing
SC thickness	Thinner vs. FT, adult	Thinner vs. adult
Microbiome	Less bacterial diversity than FT; decrease in richness followed by an increase	More diverse than PT
NMF level	Lower vs. adult, comparable to FT initially then increasing to higher than adult	Lower vs. adult, comparable to PT initially then increasing to higher than adult
Filaggrin (FLG) (stratum corneum)	Lower vs. FT at birth, higher vs. adults at birth and 2–3 months later	Higher vs. adults at birth and 2–3 months later
Filaggrin processing proteins (SC)^a^	Higher vs. adult 2–3 months after birth	Higher vs. adult 2–3 months after birth
Protease inhibitors/enzyme regulators (SC)^b^	Higher vs. adult at birth and 2–3 months later	Higher vs. adult at birth and 2–3 months later
Antimicrobial proteins (SC)^c^	Higher vs. adult 2–3 months after birth	Higher vs. adult 2–3 months after birth

a Filaggrin processing biomarkers: FLG, FLG2, CASP14, ASPRV1, TGM1.

b Protease inhibitors/enzyme regulators: PI3, SERPINB3, SERPINB4.

c Antimicrobial proteins: S100A7, S100A8, S100A9, LFT, MPO.

Overexpression of ECM genes in infant skin versus adult skin suggests their importance in newborn skin adaptation. ECM components influence cell proliferation, adhesion, apoptosis (Daley et al., 2008), barrier repair (Sumigray and Lechler, 2015) and connect the epidermis and dermis for tissue integrity. The rate of ECM modification and renewal is high in wound healing and response to infection (Sumigray and Lechler, 2015). ECM organization and structure organization were increased in infant skin. The ADAM9 gene, for example, produces MMP9 and facilitates wound healing by regulating keratinocyte migration collagen VII shedding (Mauch et al., 2010). Fetal skin demonstrates rapid and scarless wound healing and differs in inflammation, cytokine response, and ECM composition versus adult skin (Hu et al., 2018). We observed increased expression of fibronectin genes FNDC3B and FNDC3A. GNDC1, FLRT2, and FLRT3 in infants. With injury or infection, immune cells produce enzymes (e.g., MMPs, ADAMs, ADAMTSs) that promote inflammation (Tomlin and Piccinini, 2018). Collagen, laminin, and fibronectin, bind to microorganisms that can degrade the ECM (Arora et al., 2021). Immune cells control ECM synthesis, assembly, remodeling, and degradation, and respond to infection (Bhattacharjee et al., 2019).

For infants, the reduced expression for keratin genes, including KRT2, KRT25, KRT27, and KRT31, that provide structural integrity, for late cornified envelope genes, including LCE1C, LCE1D, LCE1 E, LCE1F, LCE2B, and for hair adaptation, that is, KRTAP genes (Khan I. et al., 2014), indicate the aspects of the epidermal barrier that develop over time after birth.

At birth, the newborn infant is equipped to survive and flourish, despite entering a vastly different environment replete with microbes, potential irritants, and high oxygen tension. While newborn skin is considered adaptive, the environmental exposures immediately after birth may alter the “intended” programmed trajectory resulting in aberrant skin or diseased states. Immune functions provided by the epidermis are, arguably, never more essential that at birth, particularly for infants born prematurely with underdeveloped skin. Gene expression in infant skin increased for processes including extracellular matrix and development while adults had increased gene expression for epidermal homeostasis and antigen processing/presentation of immune function. Newborn infant stratum corneum contained protease inhibitors/enzyme regulators to interact with microorganisms and moderate desquamation to ensure a barrier. Antibacterial proteins were higher in infants compared to adults well after birth, suggesting a role in immune function.

In many cases, the literature on early epidermal immunity is limited to descriptive information. Clearly, additional research is needed to delineate the gaps in our current knowledge of skin development, particularly regarding the rates of change in the epidermal barrier adaptation process as a function of gestational age. It does not yet include information among the most premature infants, names those of 22–28 gestational age. As the limit of viability decreases, the need to implement clinical practices to facilitate epidermal maturation and effective function become essential. Further research is important to understand the factors, including environmental conditions, microbiome development, and skin-device interactions, that trigger specific phenotypes, for example, atopic dermatitis, during gestation, and following birth. Reduction in neonatal mortality and morbidity is a global priority and a challenge that requires multiple research and clinical specialties. The continuous advances in research tools of genomics, proteomics, metabolomics, and bioinformatics and the availability of precious data from repositories will prime the research system for further advances. The present, available results collectively serve to guide clinical practice and the implementation of strategies to facilitate robust infant barrier integrity and function.
