# Supplementary material for: Epidermal Immunity and Function: Origin in Neonatal Skin
Source: Front Mol Biosci. 2022 Jun 8;9:894496. doi: 10.3389/fmolb.2022.894496 (PMC9215705; doi:10.3389/fmolb.2022.894496)
Supplement: Supplementary file 2 [file Table2.DOCX]

**Supplementary Table S2.** Top Gene Ontology themes increased for adults versus infants with adjusted p value <=0.0001

| **ID** | **Description** | **Count** | **pvalue** | **p.adjust** |
| --- | --- | --- | --- | --- |
|  | **Biological Process** |  |  |  |
| GO:0008544 | epidermis development | 51 | 1.99E-29 | 3.48E-26 |
| GO:0043588 | skin development | 49 | 1.01E-29 | 3.48E-26 |
| GO:0030216 | keratinocyte differentiation | 42 | 1.42E-28 | 1.65E-25 |
| GO:0031424 | keratinization | 36 | 4.69E-27 | 4.09E-24 |
| GO:0009913 | epidermal cell differentiation | 43 | 1.4E-26 | 9.79E-24 |
| GO:0030855 | epithelial cell differentiation | 46 | 8.42E-16 | 4.9E-13 |
| GO:0070268 | cornification | 19 | 8.88E-15 | 4.42E-12 |
| GO:0018149 | peptide cross-linking | 15 | 1.45E-14 | 6.34E-12 |
| GO:0019884 | antigen processing and presentation of exogenous antigen | 19 | 3.35E-11 | 1.3E-08 |
| GO:0002478 | antigen processing and presentation of exogenous peptide antigen | 18 | 1.38E-10 | 4.82E-08 |
| GO:0019882 | antigen processing and presentation | 20 | 1.79E-10 | 5.67E-08 |
| GO:0048002 | antigen processing and presentation of peptide antigen | 18 | 5.31E-10 | 1.54E-07 |
| GO:0050778 | positive regulation of immune response | 31 | 1.35E-08 | 3.62E-06 |
| GO:0002484 | antigen processing and presentation of endogenous peptide antigen via MHC class I via ER pathway | 5 | 1.98E-08 | 4.62E-06 |
| GO:0002486 | antigen processing and presentation of endogenous peptide antigen via MHC class I via ER pathway, TAP-independent | 5 | 1.98E-08 | 4.62E-06 |
| GO:0002250 | adaptive immune response | 24 | 2.53E-08 | 5.51E-06 |
| GO:0019883 | antigen processing and presentation of endogenous antigen | 7 | 3.26E-08 | 6.69E-06 |
| GO:0002684 | positive regulation of immune system process | 38 | 4.13E-08 | 8.01E-06 |
| GO:0002476 | antigen processing and presentation of endogenous peptide antigen via MHC class Ib | 5 | 5.22E-08 | 9.59E-06 |
| GO:0002443 | leukocyte mediated immunity | 33 | 8.85E-08 | 1.54E-05 |
| GO:0002428 | antigen processing and presentation of peptide antigen via MHC class Ib | 5 | 1.16E-07 | 1.76E-05 |
| GO:0002475 | antigen processing and presentation via MHC class Ib | 6 | 1.06E-07 | 1.76E-05 |
| GO:0002480 | antigen processing and presentation of exogenous peptide antigen via MHC class I, TAP-independent | 5 | 1.16E-07 | 1.76E-05 |
| GO:0060333 | interferon-gamma-mediated signaling pathway | 11 | 1.26E-07 | 1.83E-05 |
| GO:0006959 | humoral immune response | 17 | 1.83E-07 | 2.46E-05 |
| GO:0050776 | regulation of immune response | 35 | 1.83E-07 | 2.46E-05 |
| GO:0019886 | antigen processing and presentation of exogenous peptide antigen via MHC class II | 11 | 2.52E-07 | 3.25E-05 |
| GO:0002495 | antigen processing and presentation of peptide antigen via MHC class II | 11 | 3.49E-07 | 4.35E-05 |
| GO:0002504 | antigen processing and presentation of peptide or polysaccharide antigen via MHC class II | 11 | 3.88E-07 | 4.67E-05 |
| GO:0001914 | regulation of T cell mediated cytotoxicity | 7 | 7.03E-07 | 8.18E-05 |
|  | **Cell Component** |  |  |  |
| GO:0001533 | cornified envelope | 22 | 3.13E-24 | 1.15E-21 |
| GO:0042611 | MHC protein complex | 14 | 2.5E-20 | 4.6E-18 |
| GO:0071556 | integral component of lumenal side of endoplasmic reticulum membrane | 12 | 2.23E-15 | 2.04E-13 |
| GO:0098553 | lumenal side of endoplasmic reticulum membrane | 12 | 2.23E-15 | 2.04E-13 |
| GO:0098576 | lumenal side of membrane | 12 | 1.71E-14 | 1.26E-12 |
| GO:0042613 | MHC class II protein complex | 9 | 8.77E-14 | 5.36E-12 |
| GO:0012507 | ER to Golgi transport vesicle membrane | 12 | 5.99E-11 | 3.14E-09 |
| GO:0009986 | cell surface | 39 | 2.34E-10 | 1.07E-08 |
| GO:0005882 | intermediate filament | 18 | 7.41E-10 | 3.02E-08 |
| GO:0045095 | keratin filament | 12 | 4.97E-09 | 1.82E-07 |
| GO:0030134 | COPII-coated ER to Golgi transport vesicle | 12 | 9.94E-09 | 3.15E-07 |
| GO:0030660 | Golgi-associated vesicle membrane | 13 | 1.03E-08 | 3.15E-07 |
| GO:0045111 | intermediate filament cytoskeleton | 18 | 1.25E-08 | 3.53E-07 |
| GO:0062023 | collagen-containing extracellular matrix | 23 | 5.93E-08 | 1.56E-06 |
| GO:0098552 | side of membrane | 26 | 6.84E-08 | 1.67E-06 |
| GO:0030057 | desmosome | 7 | 7.74E-08 | 1.75E-06 |
| GO:0030662 | coated vesicle membrane | 15 | 8.08E-08 | 1.75E-06 |
| GO:0042612 | MHC class I protein complex | 5 | 1.05E-07 | 2.15E-06 |
| GO:0030669 | clathrin-coated endocytic vesicle membrane | 8 | 1.55E-07 | 0.000003 |
| GO:0045334 | clathrin-coated endocytic vesicle | 9 | 2.2E-07 | 4.03E-06 |
| GO:0072562 | blood microparticle | 11 | 4.87E-07 | 8.46E-06 |
| GO:0030658 | transport vesicle membrane | 15 | 5.07E-07 | 8.46E-06 |
| GO:0030139 | endocytic vesicle | 18 | 6.4E-07 | 1.02E-05 |
| GO:0010008 | endosome membrane | 23 | 8.54E-07 | 1.31E-05 |
| GO:0030666 | endocytic vesicle membrane | 13 | 1.26E-06 | 1.84E-05 |
| GO:0031012 | extracellular matrix | 24 | 1.44E-06 | 2.04E-05 |
| GO:0005798 | Golgi-associated vesicle | 13 | 2.33E-06 | 3.17E-05 |
| GO:0030176 | integral component of endoplasmic reticulum membrane | 12 | 2.65E-06 | 3.47E-05 |
| GO:0031227 | intrinsic component of endoplasmic reticulum membrane | 12 | 4.66E-06 | 5.89E-05 |
| GO:0030665 | clathrin-coated vesicle membrane | 10 | 7.77E-06 | 9.51E-05 |
|  | **Molecular Function** |  |  |  |
| GO:0042605 | peptide antigen binding | 10 | 7.53E-13 | 3.85E-10 |
| GO:0005198 | structural molecule activity | 37 | 1.12E-11 | 2.86E-09 |
| GO:0003823 | antigen binding | 11 | 1.41E-09 | 2.41E-07 |
| GO:0042277 | peptide binding | 20 | 9.4E-09 | 1.2E-06 |
| GO:0033218 | amide binding | 20 | 2.28E-07 | 2.33E-05 |
